# 10 kHz Shifted-Excitation Raman Difference Spectroscopy with Charge-Shifting Charge-Coupled Device Read-Out for Effective Mitigation of Dynamic Interfering Backgrounds

**DOI:** 10.1177/00037028231167441

**Published:** 2023-04-25

**Authors:** Sara Mosca, Kay Sowoidnich, Megha Mehta, William H. Skinner, Benjamin Gardner, Francesca Palombo, Nicholas Stone, Pavel Matousek

**Affiliations:** 1 Central Laser Facility, Research Complex at Harwell, STFC Rutherford Appleton Laboratory, UKRI, Harwell Campus OX11 0QX, UK; 2 Ferdinand-Braun-Institut, Leibniz-Institut für Höchstfrequenztechnik, Gustav-Kirchhoff-Str. 4, 12489 Berlin, Germany; 3 Department of Physics and Astronomy, 3286 University of Exeter, Exeter EX4 4QL, UK

**Keywords:** Raman spectroscopy, charge-coupled device, CCD, ambient light interference, charge-shifting, shifted excitation Raman difference spectroscopy, SERDS, fluorescence interference

## Abstract

In this work we demonstrate an advanced concept of a charge-shifting charge-coupled device (CCD) read-out combined with shifted excitation Raman difference spectroscopy (SERDS) capable of operating at up to 10 kHz acquisition rates for the effective mitigation of fast-evolving interfering backgrounds in Raman spectroscopy. This rate is 10-fold faster than that achievable with an instrument we described previously and is overall 1000-fold faster than possible with conventional spectroscopic CCDs capable of operating at up to ∼10 Hz rates. The speed enhancement was realized by incorporating a periodic mask at the internal slit of an imaging spectrometer permitting a smaller shift of the charge on the CCD (8 pixels) to be required during the cyclic shifting process compared with the earlier design which employed an 80-pixel shift. The higher acquisition speed enables the more accurate sampling of the two SERDS spectral channels, enabling it to effectively tackle highly challenging situations with rapidly evolving interfering fluorescence backgrounds. The performance of the instrument is evaluated for heterogeneous fluorescent samples which are moved rapidly in front of the detection system aiming at the differentiation of chemical species and their quantification. The performance of the system is compared with that of the earlier 1 kHz design and a conventional CCD operated at its maximum rate of 5.4 Hz as previously. In all situations tested, the newly developed 10 kHz system outperformed the earlier variants. The 10 kHz instrument can benefit a number of prospective applications including: disease diagnosis where high sensitivity mapping of complex biological matrices in the presence of natural fluorescence bleaching restricts achievable limits of detection; accurate data acquisition from moving heterogeneous samples (or moving a handheld instrument in front of the sample during data acquisition) or data acquisition under varying ambient light conditions (e.g., due to casting shadows, sample or instrument movement). Other beneficial scenarios include monitoring rapidly evolving Raman signals in the presence of largely static background signals such as in situations where a heterogeneous sample is moving rapidly in front of a detection system (e.g., a conveyor belt) in the presence of static ambient light.

## Graphical abstract

**Graphical Abstract:**
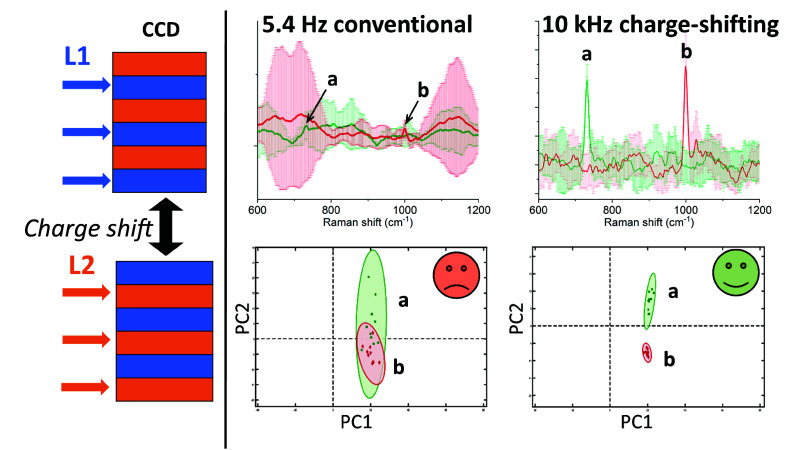
This is a visual representation of the abstract.

## Introduction

Raman spectroscopy is a powerful analytical technique that is increasingly applied to a wide range of analytical problems, often involving real-world scenarios with untreated and impure samples, and performed outside a laboratory environment under uncontrolled illumination conditions. Such measurements can involve complex sample matrices giving rise to complicated time-varying interfering spectral backgrounds, which can be present at significant levels even when using traditional near-infrared (NIR) excitation wavelengths (e.g., 785 or 830 nm) and/or measurements performed in the presence of ambient light such as in field applications. Another example is the retrieval of weak Raman signals from complex biological matrices in medical diagnosis where even a moderate level of fluorescence poses a major challenge by severely restricting the achievable limit of detection (LOD). This translates, for example, to limiting how early, or if at all, a particular disease or medical condition can be diagnosed. To mitigate these issues, numerous background suppression methods have been proposed and demonstrated with varying degree of effectiveness and instrumental complexity.^1^ Among these, fast-time gating constitutes an effective approach with moderate to high instrumental complexity.^2^ Instrumentally, a simpler approach is shifted excitation Raman difference spectroscopy (SERDS) utilizing only two continuous wave excitation wavelengths.^3,4^ However, unlike the time-gating concept, it does not facilitate the removal of photon shot noise from the detected spectra associated with the fluorescence or ambient light backgrounds,^5–7^ although it does remove effectively other artefacts from spectra associated with the presence of high-level backgrounds such as those due to charge-coupled device (CCD) etaloning and filter ripple distortions.^7^ These artefacts can often be much larger than the photon shot noise. They can also be similar in appearance to Raman bands, which makes their numerical removal challenging. Consequently, major improvements to the quality of detected Raman spectra and LODs can often be achieved by suppressing these artefacts using SERDS.

Nowadays, the SERDS technique is used relatively frequently in fluorescence challenging situations for its good balance between its relative instrumental simplicity and effectiveness. In its basic form, the method relies on performing two consecutive measurements at slightly offset excitation wavelengths (typically on the order of the bandwidth of the detected Raman bands).^8^ Since fluorescence is emitted mostly from vibrational relaxed electronic states, its spectral profile is practically unchanged between the two excitation wavelengths, whereas Raman spectra exhibit a shift in the detected spectra equal to the wavenumber difference between the two excitation wavelengths. The subtraction of these two spectra then yields a difference spectrum where the fluorescence background cancels out along with the above-mentioned associated spectral artefacts, revealing Raman bands as derivative-like features. Such spectra can then be used directly in spectral analysis^9^ or first Raman spectra in conventional form can be reconstructed from these and then analysed.^10^ The latter approach provides more intuitive, easier-to-interpret results and, for this reason, it often constitutes the most favored approach. Although it should be noted that an additional step of reconstruction of the difference spectra is an extra mathematical operation that can complicate data sets polluting them potentially with additional artificial distortions that can mask underlying chemical information contained in the original data. For this reason, using SERDS difference spectra in multivariate data analysis directly may sometime yield better, more accurate results. In an analogous manner, the SERDS method also suppresses ambient light backgrounds and artefacts stemming from its presence as this signal source is also independent of the laser excitation wavelength.^11^

The SERDS method becomes considerably less effective, however, in situations where the interfering backgrounds are non-static, evolving spectrally or intensity-wise during the time required for spectral acquisition. This is because any changes in relative intensity or spectral profile between the two SERDS spectral channels lead to imperfect cancellation of these backgrounds upon their subtraction from each other, leaving behind residual backgrounds with the emergence of associated spectral artefacts. This restricts the attainable limits of detection and accuracy of the measurements. This impacts the detectability of low-concentration analytes (e.g., ability to determine the presence or absence of disease) in complex matrices such as in medical diagnosis.^12^ Such unstable backgrounds can arise due to multiple factors; for example, they can be due to ambient light variation during the measurement caused by an operator or bystander casting a shadow, sample, or instrument movement during data acquisition or simply clouds moving in the sky and casting shadows. Other causes include fluorescence intensity or/and spectral profile variations caused by the movement of a heterogeneous sample in front of the Raman instrument (or the movement of the instrument itself). Another important and practically relevant effect is fluorescence bleaching induced by the laser excitation light. This is especially common and problematic when mapping biological samples using Raman microscopy.^12,13^ Other relevant scenarios include the retrieval of chemical information from deep layers of highly heterogeneous and moving matrices (e.g., biological tissues in medical diagnosis in vivo) using spatially offset Raman spectroscopy (SORS).^14^

Such dynamic interfering backgrounds can effectively be mitigated by the rapid, alternating sampling of the two SERDS spectral channels on a timescale much faster than the characteristic timescale of the background variation. This requires a synchronous acquisition of the Raman spectra for the corresponding two SERDS channels by the detector as the spectra are summed ultimately in each individual SERDS spectral channel and subtracted from each other. This is, however, challenging with conventional spectroscopic CCDs that are limited in their ability to collect two consecutive spectra by the speed of digitization and transfer of data to a computer – for example, with a traditional spectroscopic CCD such as DU420A-BR iDus (Andor, Oxford Instruments), this limits such readouts to <10 Hz.^9,15^ These speeds may not be sufficient to accurately map fast-evolving backgrounds one could encounter in the abovementioned challenging applications.

### Advanced Charge-Shifting CCD Method with 10 kHz SERDS Acquisition

Here, we present a method enabling us to perform such spectral sampling at 10 kHz repetition rates, i.e., more than 1000-fold faster than possible with conventional spectroscopic CCDs. The concept is an advanced variant of our previously demonstrated CCD charge-shifting (CS) method that was capable of operating with detection rates of up to 1 kHz.^9,15,16^ The basic principle of the method was described in detail previously.^9^ In brief, with the previous CS approach, only a part of a CCD array, around its center, is illuminated with the other two CCD segments (above and below, assuming the spectral dispersion is horizontal) serving as dark, unilluminated charge storage areas. This can be accomplished externally for example by inserting a mask in the image plane of an imaging spectrograph (e.g., at its entrance slit). Charge induced by the signal on the CCD is then moved periodically between the illuminated and non-illuminated zones (up and down) in synchrony with the sample illumination modulation (in the SERDS case, switching between the two laser excitation wavelengths). This leads to the accumulation of two distinct Raman spectra in two different charge zones. As no digitization takes place in this oscillation phase, acquisition rates can be much faster than that with conventional read-out where each frame has to be digitized separately. The speed is limited only by the CCD charge-shifting rate. An inherent downside of this approach is the necessity for reserving a part of the CCD for charge storage resulting in a restriction of the usable CCD area for illumination purposes. However, this limitation can be outweighed by the improvement in spectral quality and LOD by employing this detection method in challenging high-level backgrounds situations in Raman spectroscopy.

It is worth noting that such fast sampling of interfering backgrounds would not be effective with conventional CCD read-outs where each frame has to be individually read even if such high digitization speeds as those possible with the charge-shifting concept could be achieved, because of the accumulation of undesirable read-out noise imprinted on spectra in multiple digitization steps.^15^ With the charge-shifting concept, this is avoided by integrating charge on the chip without any digitization in each modulation cycle and final read-out being performed only once at the end of the measurement, after accumulating multiple shifting cycles (e.g., 100–1000 cycles).

Here, we present a new charge-shifting design incorporating a special periodic mask in the detection system and multi-track-CCD charge storage areas enabling an increase of the sampling frequency by an order of magnitude over our previous design^9,15^ and we demonstrate its advantages over the earlier variant. An additional benefit of the periodic mask concept over our previous design is the improvement of CCD coverage used for illumination and spectral acquisition, from 25%^9^ to 44%.

Recently, a similar concept utilizing the charge-shifting approach with the specialized camera system Zurich Imaging Polarimeter (ZIMPOL) was applied to Raman optical activity (ROA) and operated in synchrony with a photo-elastic modulator at 45 kHz.^17^ Another related SERDS acquisition approach was also demonstrated by Shimada et al.^12^ It employs no CCD charge movement, but instead uses deflection of Raman light for the two SERDS spectral channels on two different CCD tracks applying an opto-galvanic mirror placed in front of the detection system. This approach could operate at 200 Hz oscillation rates. Although this was effective in suppressing interfering evolving backgrounds in the context of the studied application comprising fluorescence bleaching, the use of moving mechanical devices prevents considerably higher rates to be achieved in order to deal with faster evolving interfering backgrounds. Additionally, the detection of the two SERDS spectra with the opto-galvanic mirror concept is achieved through different CCD pixels and involves somewhat different optical paths between the opto-galvanometric system and the CCD detector. This can lead to potential residual artefacts in the SERDS difference spectrum stemming from not complete equality of the two detection channels and the unavoidable presence of other spectral or imaging distortions in the detection system (e.g., spectrometer “smile” effect^18,19^). These issues are intrinsically avoided in the charge-shifting approach as the optical paths and CCD detection pixels for the two SERDS acquisition channels are identical.

## Materials and Methods

### Charge-Shifting Shifted Excitation Raman Difference Spectroscopy Setup

**Figure 1.**
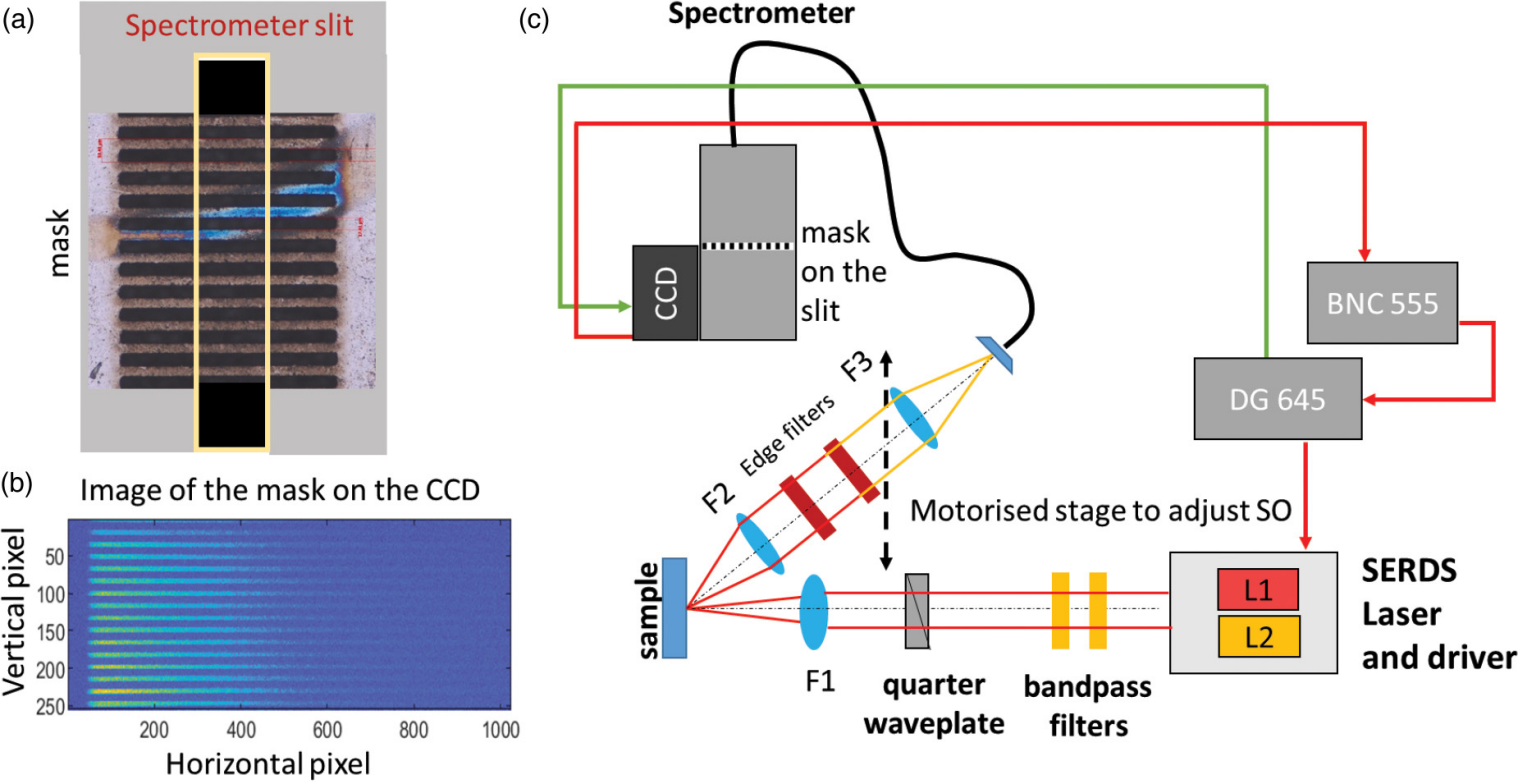
(a) Sketch of the periodic mask placed in front of the second stage slit of a Raman spectrometer. (b) Conventional 2D read-out of the CCD in presence of a spectrally broad illumination source showing the mask pattern on the sensor. (c) Schematics of the charge-shifting SERDS setup with, in the excitation path, dual-wavelength (L1, L2) SERDS laser and driver emitting light around 830 nm, bandpass filters, quarter waveplate, a 100 mm lens (F1) that focuses the laser beam on the sample surface. The collection path is assembled on a motorized stage defining the spatial offset (SO) consisting of 50 mm collection lenses (F2, F3), Raman edge filters, optical fiber bundle, spectrometer, and charge-shifting CCD. The DG645 digital delay generator and BNC model 555 digital delay generator connected to the laser driver and CCD allowed for the correct timing and synchronization of the charge-shifting detection.

A schematic diagram of the charge-shifting SERDS setup is shown in Figure 1. The device is based around a custom-built system described in a previous work.^9^ It utilizes a purpose-designed SERDS laser module^20^ emitting light at λ_1 _= 829.40 nm and λ_2 _= 828.85 nm, with both wavelengths being individually and rapidly addressable. The collimated laser beam passes through two bandpass filters (LL01-830-25, Semrock, Inc.) and a quarter waveplate (WPQ05M-830, Thorlabs) converting the two orthogonal linearly polarized laser beams into left and right circularly polarized beams. The excitation laser is focused on the sample surface using a 100 mm lens (F1 in Figure 1c) to an approximate lateral spot size of ∼700 μm diameter at the sample surface (10% to 90% transmitted power points derived from translating an obscuring sharp edge across the laser beam at the sample plane). The Raman scattered light is collected at an angle of ∼35° to the normal incidence from a zone of approximately 1.2 mm diameter on the sample surface through a telescope system made of two 50 mm lenses (F2 = F3 in Figure 1c), with two Raman longpass filters in between them to suppress residual laser light and relayed via an optical fiber bundle system to an imaging spectrometer (HoloSpec *f*/1.8i, Kaiser Optical Systems) coupled to a custom-made charge-shifting CCD (DU420A-BR-DD-9UW, Andor Technology, CCD-19689). The fiber bundle (custom-made, 22 active fibres^21^ with a core diameter of 220 μm each; CeramOptec Industries, Inc.) has a round-to-linear transformation configuration to match the spectrograph slit.^14^ The system can operate in both the conventional and SORS modes. The SORS spatial offset is achieved by moving the entire collection path assembly along the plane parallel to the sample surface (see Figure 1c) with a motorized stage (MTS25-Z8 with KDC101 controller, Thorlabs) in a range between spatial offsets (SOs) SO = 0 mm to 22 mm. The Raman measurement presented here were obtained with SO = 0 mm (near-surface collection mode) and 3 mm (sub-layer probing). The applied SORS spatial offset for individual experiments is indicated in the respective figure captions.

A laser micro-machined metal mask was engraved out of a tungsten foil (external dimension 10 mm × 5 mm, thickness = 100 μm; mask grid dimensions: height = 6 mm, width = 3 mm). The mask was placed in the optical plane of an internal spectrometer slit (Figure 1a) to shield parts of the CCD sensor as required for the CS method.^15^ The schematic of the mask is shown in Figure 1a. It takes into account the internal magnification of the spectrograph (M = 1.133) creating a pattern on the sensor shielding periodically 8-pixels (px) along the vertical axis (see Figure 1b). The specific dimensions of the projected mask pattern with respect to the CCD sensor is provided in Figure S1 (Supplemental Material) together with the experimental characterization of the contrast between the two areas (illuminated versus non-illuminated) formed on the sensor.

The timing and synchronization between the two laser wavelengths required for SERDS and the charge-shifting CCD read-out is controlled by a digital delay generator (DG645, Stanford Research Systems) connected to the laser driver module and external CCD trigger (Figure 1c). A second digital delay generator (Model 555, Berkeley Nucleonics Corporation) connected to the CCD output shutter signal port inhibits the DG645 delay generator from pulsing during the final CCD read-out and digitization phase. Further details can be found in our previous study.^9^

### Charge-Shifting Method

The working principle of the charge-shifting method was explained in detail in our previous work.^9,15^ Briefly, an external trigger signal at the desired frequency (e.g., 1 kHz or 10 kHz) starts the first cycle in which the area on the CCD chip (not shielded by the mask – i.e., illuminated area) receives the emitted signal (i.e., mixture of Raman scattered light, fluorescence light, room light or external interference) from the sample excited at the first excitation wavelength (L1) and the generated charges are accumulated in charge group 1 of the electronic register (channel 1, CH1). After time equal to the inverse of the frequency (i.e., 1 ms for 1 kHz or 0.1 ms for 10 kHz), the next trigger pulse initiates the second cycle in which all charges of the electronic register are shifted by eight vertical pixel rows, moving the charges accumulated in CH1 behind the shielded area. Subsequently, the unshielded pixels on the CCD chip are illuminated by the signal coming from the second excitation wavelength (L2) but the corresponding charges are now accumulated in a different charge group of the electronic register (CH2) (but detected through the same physical CCD pixels as signal induced by L1). The sequence of the alternate charge accumulations in the two distinct charge groups of the electronic register while illuminating the same pixels of the CCD chip is repeated a specific number of times (called “number of cycles”), corresponding to a desired total acquisition time. For example, for an external trigger frequency of 10 kHz, 50 000 cycles correspond to a 5 s overall acquisition time. In the final step, all the accumulated charges on the CCD chip are read out and digitized (as a 2D image). Using the Andor Solis software, the fastest possible option for both the vertical pixel shift (equal to 8.25 μs) and a horizontal pixel shift readout rate (100 kHz at 16-bit) was selected for both charge-shifting and for the conventional operation mode. Initially, the injection currents for both laser cavities are set to 0 mA in the laser driver module, and, during the charge-shifting acquisition, a square-wave modulation is applied at half frequency with respect to the selected operating CS frequency (i.e., 1 kHz or 10 kHz) in order to emit a specific excitation wavelength (L1 or L2) only when the charge is in the correct position of the electronic register. The voltage of the modulation is adjusted to obtain an average laser power of 52 mW at the sample surface for both alternating operated wavelengths. Moreover, to avoid laser emission during the charge movement, a pulse delay (Td) between the CCD trigger signal and the laser pulse output is applied. Laser pulse delay (Td) and duration (Tw) are adjusted in order to have the same equivalent laser exposure time in all the charge-shifting and conventional acquisition modalities (Table I).

**Table I.** Overview of experimental parameters for charge-shifting and conventional CCD read-out modalities.

Acquisition time: 5 s					
Modality	External trigger frequency (Hz)	Number of cycles equivalent to 5 s	Tw^a^ (ms)	Td (ms)	L1 or L2 time (ms)
CS	1000	5000	0.31	0.680	775
CS	10000	50000	0.031	0.068	775
Conventional	5.4	36	43.1	29	776

Acquisition time: 0.5 s					
Modality	External trigger frequency (Hz)	Number of cycles equivalent to 0.5 s	Tw (ms)	Td (ms)	L1 or L2 time (ms)
CS	1000	500	0.31	0.680	77.5
CS	10000	5000	0.031	0.068	77.5
Conventional	5.4	6	25	29	75

^a^ Tw = laser pulse width, Td = laser pulse delay, L1 or L2 time indicates the actual overall illumination time for each laser source at the sample for two different acquisition times of 5 s and 0.5 s used in the study.

### Conventional Read-Out

For comparison, SERDS spectra were also recorded in conventional SERDS CCD read-out mode. The CCD was externally triggered at a frequency of 5.4 Hz by the DG645 delay generator to record a kinetic series using sub-acquisition times of 100 ms and full vertical binning modality (adjacent spectra correspond to excitation at L1 and L2).^9^ Laser pulse delay (Td) and duration (Tw) were adjusted to match the illumination conditions used in charge-shifting mode (see Table I). For example, 36 acquisitions and a laser pulse width (Tw) of 43.1 ms were used to match the equivalent exposure time used for the 5 s CS modalities, while 6 acquisitions and a laser pulse width (Tw) of 25 ms were used to match the equivalent exposure time used for the 0.5 s.

### Samples

For the qualitative investigations aiming at the differentiation of chemical species, two blocks of turbid media were used as sub-layer model samples for the first set of SERDS experiments: polytetrafluoroethylene (PTFE) and polystyrene (PS) with a dimension in mm (*x*,*y*,*z*) = (50,50,12).^22^ On top of the two model plastics, a heterogeneous fluorescent label was added to mimic a challenging interfering fluorescence emanating from a surface layer. This was done by creating an irregular, heterogeneous pattern with an IR-125, Rhodamine-700 and 3,3'-diethyloxadicarbocyanine iodide (DODCI) laser dye solutions (Lambdachrome Laser Dyes) on top of a thin optical cleaning tissue (MC-5 Thorlabs). Once the pattern was dry, a transparent tape was used to attach the label to the model plastic (picture shown in results section, Figure 3). For each model sample, 10 repeat spectra were recorded with each method (charge-shifting mode at 10 kHz, 1 kHz, and conventional read-out at 5.4 Hz).

The second set of measurements was performed through a glass vial (6 mL total volume, with an external diameter of 15 mm and a height of 55 mm) containing a mixture of ethanol (E) and methanol (M) solutions (encoded as follows E50M50 = ethanol 50%, methanol 50%). Twelve selected concentrations were analyzed in the same glass vial in the following mixtures: E100M0, E90M10, E80M20, E70M30, E60M40, E50M50, E40M60, E30M70, E25M75, E20M80, E10M90, E0M100. To ensure reproducible sample conditions, the same vial was used for all the measurements, and it was thoroughly cleaned with distilled water and dried with tissue paper between filling the different alcohol solutions. A similar fluorescent label as used for the qualitative investigations was coupled to the vial used for the measurements by painting individually with different patterns the three laser dyes mentioned above (IR-125, Rhodamine-700 and DODCI) on top of a transparent thin plastic sheet (picture in Figure S6a, Supplemental Material). For each concentration, eight repeat spectra were recorded in the same vial with each method (charge-shifting mode at 10 kHz, 1 kHz, and conventional read-out at 5.4 Hz).

Figure S2 (Supplemental Material) shows the conventional Raman spectra of all model samples used in this work, collected with the excitation wavelength L1 (λ_1_ = 829.40 nm), highlighting the main Raman bands used for further analysis. In both sets of measurements, SERDS spectra were acquired with a fixed spatial offset of SO = 3 mm, while the spatially heterogeneous sample was moved perpendicular to the excitation laser beam (with the help of mechanical guidance) at moderate speed in an irregular pattern to mimic a sample movement and the related variation of fluorescence background.

### Data Analysis

**Figure 2.**
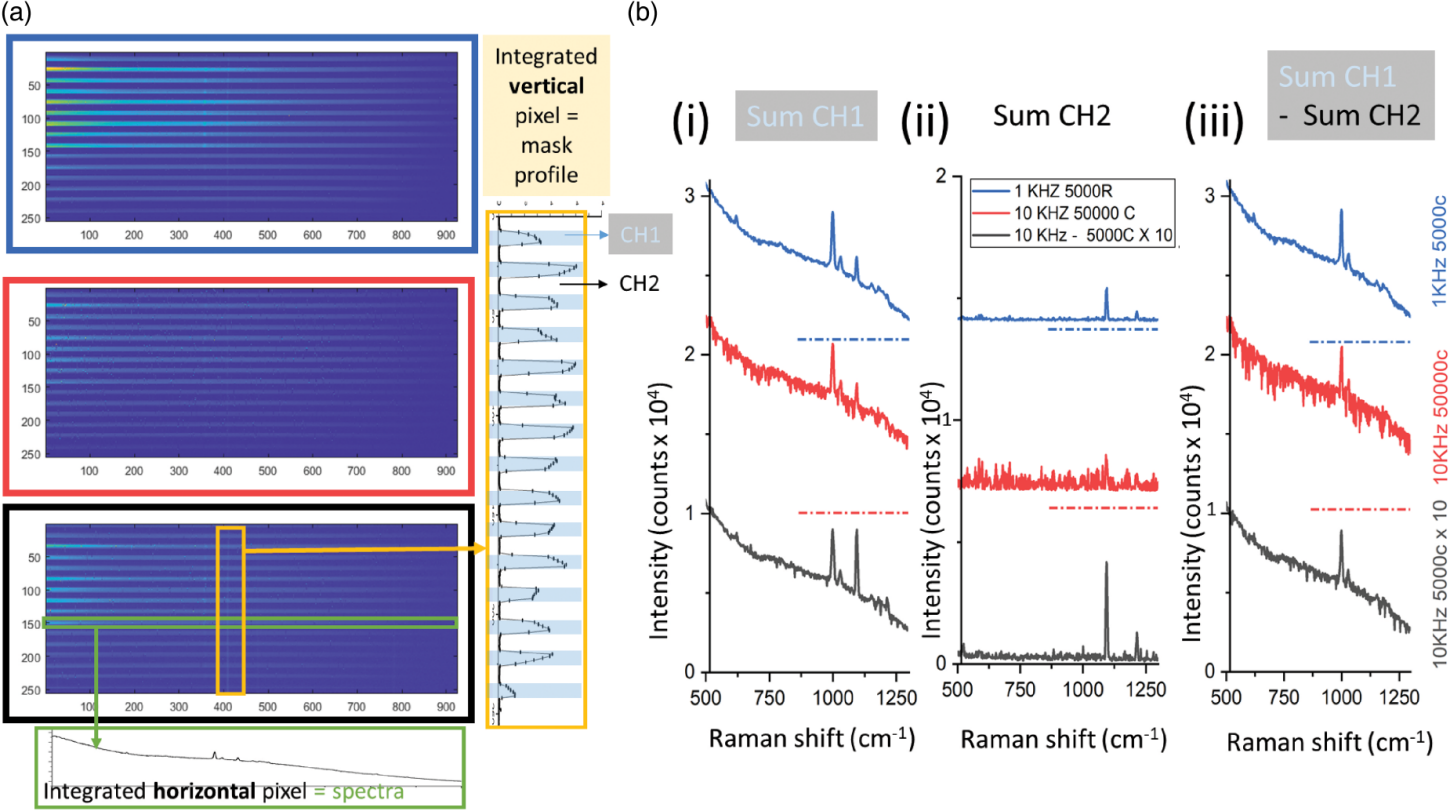
(a) CS 2D image read-out at 1 kHz and 10 kHz with different numbers of charge-shifting cycles performed on a moderately fluorescent layer on top of a PS layer: 1 kHz at 5000 cycles (blue rectangle), 10 kHz at 50 000 cycles (red rectangle), 10 repetitions at 10 kHz at 5000 cycles (black rectangle). Green and yellow rectangles on the two-dimensional (2D) read-out show respectively the results of horizontal and vertical integration of charges. The former was used to derive the contrast between CH1 and CH2 and the latter to extract the spectra of CH1 and CH2. (b) Raman spectra were obtained in the 3 different conditions by summing (i) CH1, (ii) CH2 and (iii) CH1–CH2. Illumination condition: CH1: L1 + room light, CH2: Room light (L2 OFF). The sample was not moving during the acquisition. The dash-dotted horizontal lines indicate the 0 counts level for the vertical shifted curves.

For the conventional SERDS read-out, the individual spectra (recorded with full vertical binning mode) for the same excitation wavelength were summed in order to obtain two single spectra for each excitation wavelength corresponding to CH1 (sum of all spectra collected with L1) and CH2 (sum of all spectra collected with L2). For the CS read-out, the full 2D-image acquisitions were processed using Matlab R2019b in order to extract the two spectra corresponding to CH1 and CH2. Specifically, the intensity along the vertical axes (256 pixels) was integrated with a fixed pattern (a rectangular shape with spatial distance/period = 8 px and integration width = 7 px; see schematic in Figure S1) optimized in order to match the image of the mask on the CCD chip. The 32 individual spectra obtained (i.e., 16 for each excitation wavelength-alternated L1, L2, L1, L2…) were summed in order to obtain two single spectra, one for each excitation wavelength corresponding to CH1 and CH2. An example of the post-processing performed on the 2D image read-out of the CS measurement with only one channel illuminated (CH1) is shown in Figure 2.

For all the acquisitions in all the read-out modalities (CS and conventional), the SERDS difference spectra were obtained by simply subtracting the two Raman spectra related to the two different excitation wavelengths captured in CH1 and CH2, respectively. In addition, reconstructed SERDS spectra were also calculated using a simple reconstruction algorithm.^23^

Multivariate analysis, i.e., principal component analysis (PCA), partial least squares regression (PLS), and partial least squares discriminant analysis (PLS-DA), were performed using SOLO software (2019 Eigenvector Research, Inc.) on both the difference and the reconstructed SERDS spectra in order to evaluate the capability of each read-out modality to differentiate between chemicals and to predict the concentration of chemical components in the presence of a strong and rapidly time-evolving fluorescence background. A simple pre-processing routine was applied using SOLO to the acquired dataset before performing PCA, PLS-DA and PLS regression analysis consisting of a seventh-order polynomial subtraction, Savitzky-Golay smoothing (five-point window) and standard normal variate (SNV) normalization in the selected spectral range (e.g., 350 to 1400 cm^–1^). In particular, PLS-DA analysis was used to evaluate the sensitivity and specificity of each read-out modality to discriminate between chemicals. Moreover, for PLS analysis, an automatic randomized algorithm (i.e., Kennard-Stone) was used to split the 96 spectra (12 concentrations × 8 repetitions) acquired for each frequency into calibration (66% of the total data) and validation (34% of the total data) sub-datasets in order to perform the calibration model and evaluate its prediction performance. To characterise the accuracy on the calibration sub-data set, a leave-one-out cross-validation approach was used within the PLS analysis.

## Results and Discussion

### Validation of 10 kHz Charge-Shifting Setup Performance

The performance of the charge-shifting SERDS system was initially tested on a simple, static sample (polystyrene, or PS) exhibiting a negligible level of fluorescence to characterise its basic performance. The tests were performed in SERDS configuration with a 0 mm spatial offset at an equivalent acquisition time of 5 s for all detection modalities. Using the charge-shifting read-out, 10 kHz with 50 000 cycles of acquisitions and 1 kHz with 5000 cycles were evaluated. For comparison, spectra were also recorded at 5.4 Hz with a conventional CCD read-out (see Table I). Figures S3a,b (Supplemental Material) shows the SERDS difference spectra as well as the reconstructed SERDS spectra (Figures S3c,d) measured under these conditions both with the room lights on and off. The plots also show a standard Raman spectrum obtained by plotting only one channel from the SERDS measurement at the bottom. These spectra illustrate the position and magnitude of interfering ambient light signals. The data evidence that all the acquisition approaches yielded SERDS spectra with good suppression of the ambient light as this was not evolving in time in terms of intensity and spectral profile during the acquisitions. Under these conditions with static low-intensity backgrounds, the conventional read-out yielded the highest signal-to-noise ratio (S/N) in the detected spectra. This is mainly attributed to noise-like features appearing in the charge-shifting detection system discussed below and associated with the charge movement on the CCD and its occasional leakage away from its “correct” location. Additionally, the higher quality of the conventional SERDS spectra is also in part contributed to the full vertical binning mode deployed in the conventional read-out yielding a lower read-out noise level overall. Corresponding reconstructed SERDS spectra are also shown and are comparable for all the detection methods. The noise-like features are considerably reduced in the reconstructed SERDS spectra. This observation is in accordance with a previous report comparing the S/Ns of SERDS difference and reconstructed spectra.^24^

Further tests performed on an opaque PTFE sample with low fluorescence background, involved increasing the number of cycles from 5000 to 50 000 to assess potential limitations of the method. This revealed the presence of a gradual degradation of charge pixel fidelity in the acquired signals with increasing number of cycles used. This is notable in Figure S4 (related to CS 10 kHz 50 000 repetitions, Supplemental Material), in the zoomed-in section where charge pockets highlighted with arrows appear to cluster locally within a specific single zone from its vertical neighborhood (the direction of the charge-shifting). The corresponding measurement was performed with one SERDS laser wavelength switched off to emphasize this effect. Testing different numbers of cycles revealed that this appears to occur at a given, relatively low probability in a step-like fashion for each location as a function of increasing number of cycles. The charges first appear to be well confined to each individual local zone but, after a certain number of cycles (and this number varies from acquisition to acquisition and from zone to zone), the charges suddenly coalesce vertically (in the direction of the charge movement) and bunch together within one pixel from a local neighborhood. As this effect can act across our mask boundaries, in effect this leads to charge leaking from one SERDS acquisition spectral zone (e.g., CH1) into the other one (e.g., CH2).

This charge leaking effect manifests itself ultimately as noise-like features appearing in the spectra (blue lines, Figures S4a,b, Supplemental Material), as also evident in Figure 2 where only one laser wavelength was active during acquisition to highlight this effect. The dark detection channel corresponding to the inactive SERDS laser wavelength shows the presence of noise due to charge leaking into this detection channel vertically (Figure 2b(ii) red line, 10 kHz at 50 000 cycles). Likewise, the detection channel corresponding to the active SERDS laser wavelength (Figure 2 b(i)) shows similar looking noise features protruding only below the spectral baseline; these are due to the absence of charges that leaked from the illuminated channel into the dark channel. The noise features appear partially reproducible as only some zones are most prone to exhibit this effect. However, the number of cycles required for a particular pixel to show this charge leak varies statistically. In essence, it is as if there was a certain probability for each charge zone for this to occur, which varies from zone to zone. For this reason, there may be scope for partial correction of this effect as a similar pattern appears after repeated measurements. However, in this study, we have not explored this strategy and the leakage effect itself was not further investigated as it was mitigated by reducing the number of charge cycles as outlined below.

As the charge leakage effect becomes significant above ∼5000 cycles, we have restricted the number of cycles to 5000 yielding acceptable results (see Table I for parameters corresponding to 0.5 s equivalent acquisition time). For investigations requiring longer acquisition times, repeated measurements and summing of these provides a better, acceptable strategy to mitigating this effect. The start of a new acquisition refreshes the CCD to its starting condition with no charges initially leaked. Consecutively running multiple sub-acquisitions, each restricted to 5000 cycles and starting with a clean condition of the electronic register, yielded better outcomes than a single long one with an equal number of overall cycles. This is illustrated in Figure 2b, where the center (red curves) and bottom (black curves) spectra show 10 kHz measurements on a sample exhibiting a moderate level of fluorescence (static measurement performed on a sample area with a low concentration of Rhodamine-700 on top of a PS block). Displayed are spectra obtained in a single acquisition with 50 000 cycles (center) and spectra obtained using 10 repeated measurements on the same sample area summed up numerically in a post-acquisition processing step, with 5000 cycles each (bottom). The latter exhibits higher spectral quality with a much lower number of charge leakage noise-like features present.

### Chemical Species Differentiation

**Figure 3.**
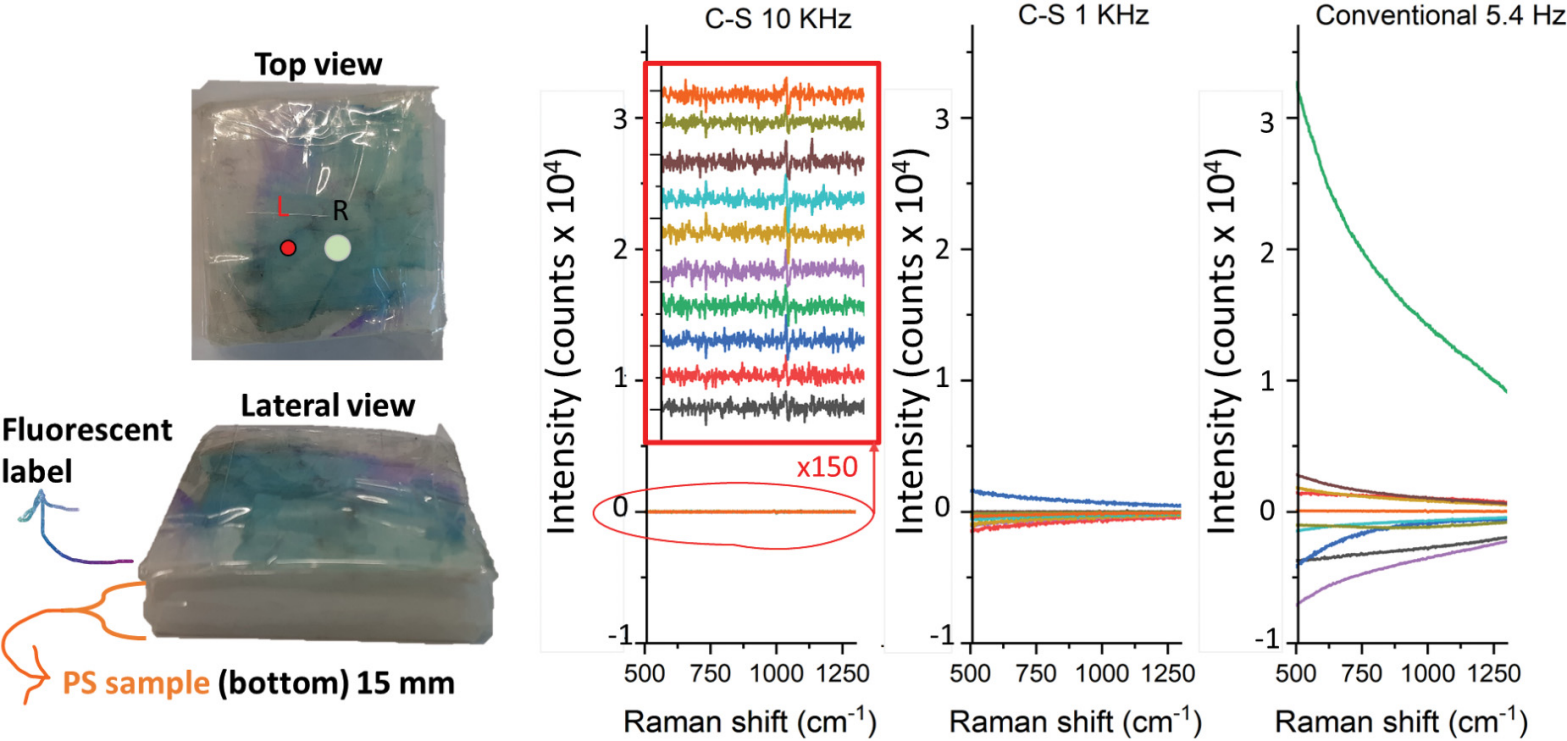
View of the model system with the highly heterogeneous fluorescent label on top of the ps sample. All the 10 SERDS difference spectra were acquired with the CS and conventional readout using SO = 3 mm.

In the next set of measurements, we looked at the performance of the 10 kHz charge-shifting system under a highly challenging situation involving fast dynamically evolving, intense fluorescence backgrounds. This was achieved using plastic samples (PS and PTFE) overpainted with a highly fluorescent agent in an irregular manner. The samples were moved rapidly by hand within the detection plane of the Raman system to induce fast spectral variations of the backgrounds. This set of experiments was performed with room light on, adding another challenge to data acquisition. The performance of the 10 kHz charge-shifting SERDS read-out was compared with that of 1 kHz operation and also with a conventional SERDS read-out where CCD signal was read individually at 5.4 Hz. The key objective of these experiments was to test the ability of each acquisition modality to differentiate the two plastic samples from each other in presence of highly challenging fast evolving backgrounds. The spectra were acquired under equal laser illumination conditions (see Table I) ensuring that a comparable number of Raman photons was detected in each experiment to enable a relative comparison of the three different approaches in terms of spectra quality. The resulting raw SERDS difference spectra, i.e., without any baseline correction applied, for all the 10 repetitions are shown in Figure 3 for the PS bottom layer sample. The conventional CCD read-out (5.4 Hz) resulted in highly fluctuating backgrounds as the SERDS spectral channels were not sampled sufficiently fast for the dynamic background to be eliminated effectively. The 1 kHz charge-shifting SERDS performed considerably better (over 30-fold). The best performance, by far, was achieved with the 10 kHz charge-shifting SERDS that exhibited practically no residual baseline distortions, with a flat baseline reproducibly present when plotted on the same scale. The background distortions were not visible even after being zoomed out ×150 as shown in the insert (where the individual spectra are vertically offset for better readability). This indicates that the 10 kHz sampling rate was sufficiently fast to effectively map the fast-evolving backgrounds equally in the two SERDS detection channels.

**Figure 4.**
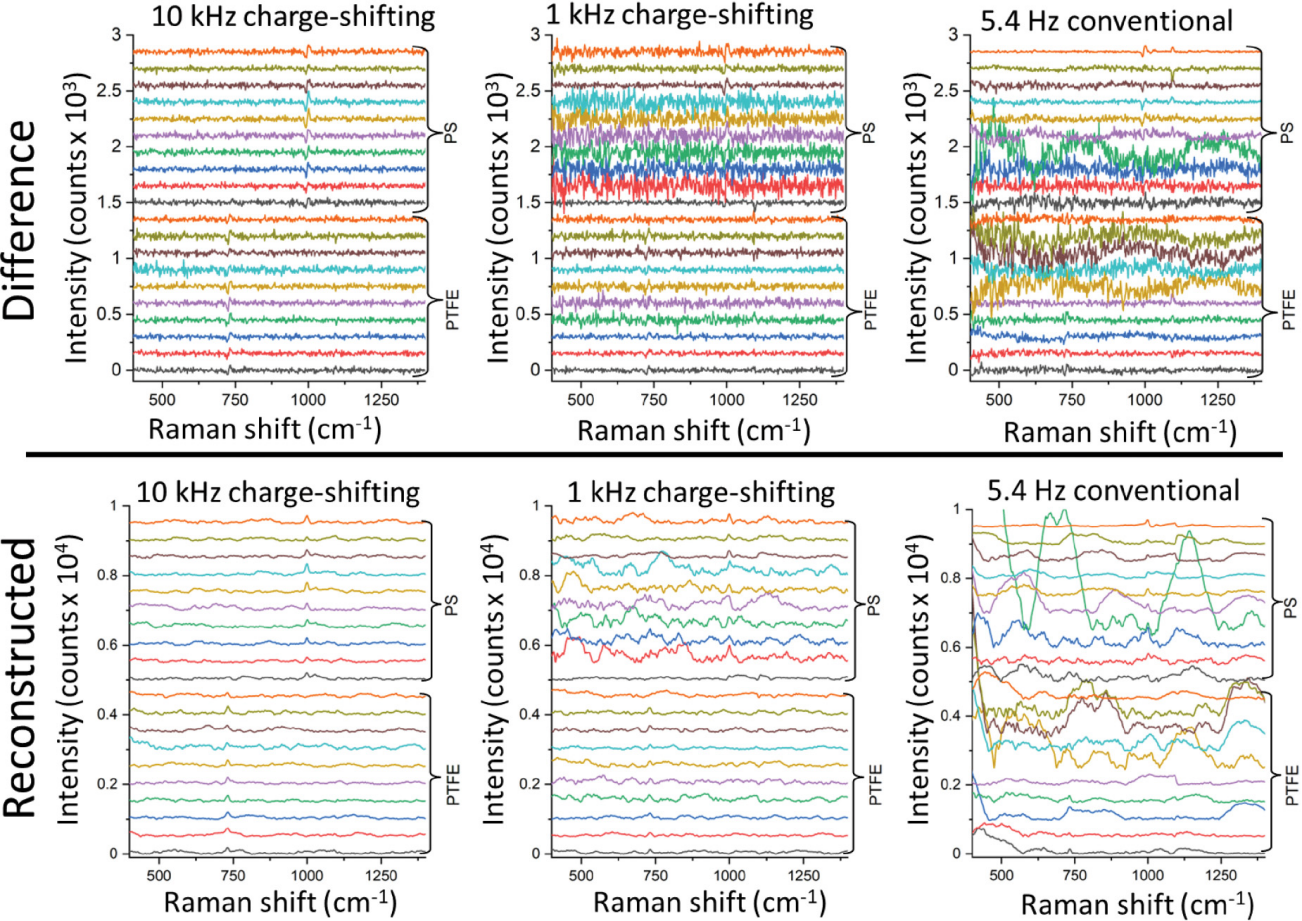
Top panel: SERDS difference spectra with the post-processing approach (background subtraction using a seventh-order polynomial). Bottom panel: reconstructed SERDS spectra from post-processed difference spectra for each read-out modality.

The SERDS difference spectra were then pre-processed by subtracting a seventh-order polynomial baseline, a simple processing step that can readily be performed without sophisticated data analysis software. The resulting spectra for both PS and PTFE samples are shown in Figure 4. Although much higher spectral stability is present here for all the three configurations, it is still evident that the 10 kHz acquisition yields superior spectra to those for the 1 kHz modality, which in turn is still much better than the conventional 5.4 Hz acquisition mode in terms of residual background noise and fluctuations between individual spectra. The entire SERDS difference spectral dataset was also numerically reconstructed using a simple reconstruction algorithm to yield SERDS spectra in conventional form. The result of this reconstruction is shown in Figure 4 in the bottom segment for both the PS and PTFE samples. Again, a similar trend is present with the 10 kHz variant yielding considerably superior spectra to both the 1 kHz and 5.4 Hz acquisition modalities. In particular, for the 5.4 Hz acquisition, residual baseline modulations present in the SERDS difference spectra lead to pronounced artefacts in the reconstructed spectra, complicating sample identification.

**Figure 5.**
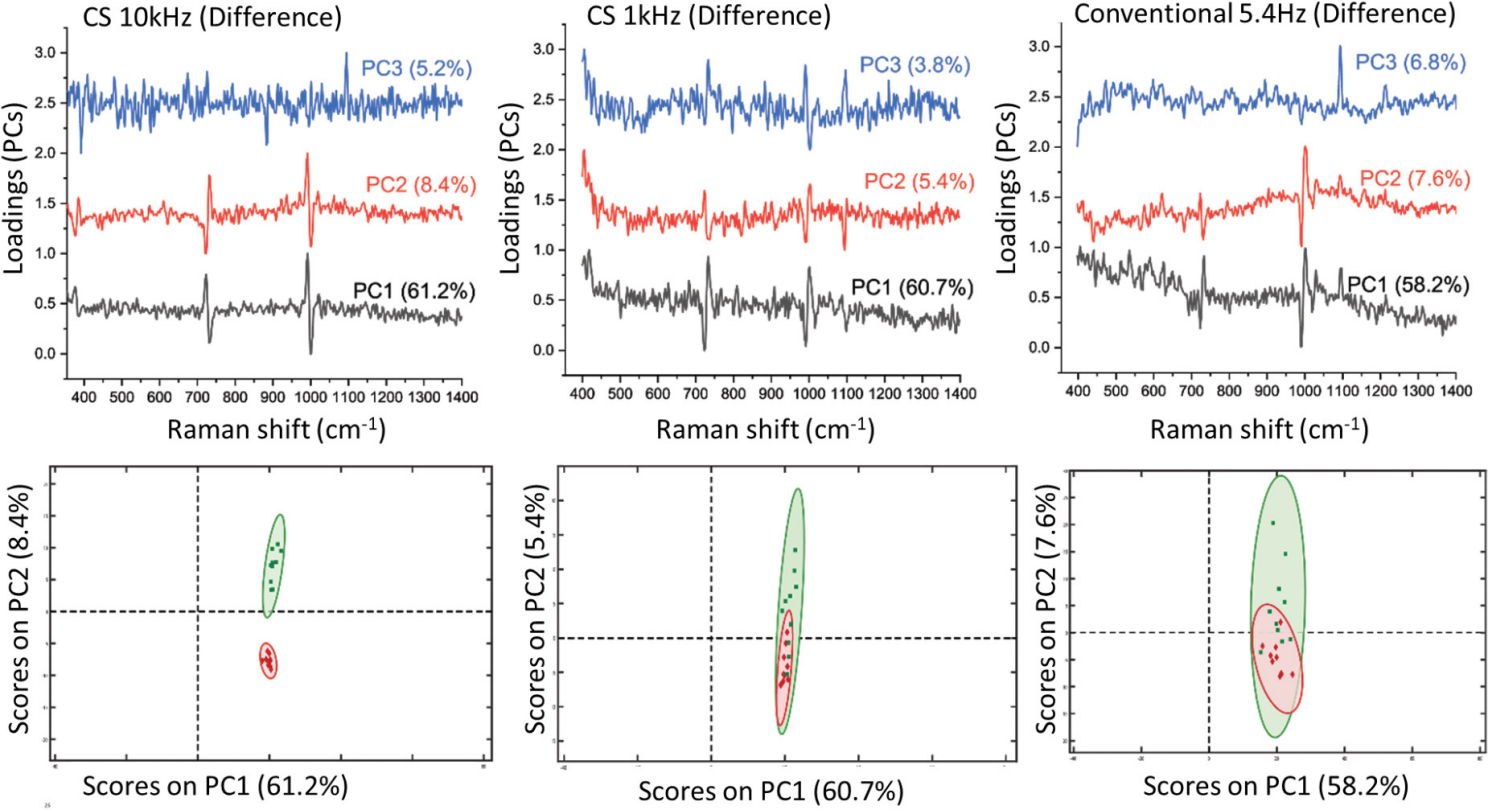
PCA results derived from SERDS difference spectra of PS and PTFE in the presence of a strong heterogeneous fluorescent signal from top layer using the three read-out modalities. Top panel: PCA loadings; bottom panel: PCA biplots.

To test the ability of the individual acquisition approaches to differentiate between the two plastics, different repetitions (i.e., 10 individual spectra for each plastic, 20 spectra in total, see Figure 4) were individually processed by principal component analysis. The results of this analysis when using SERDS difference spectra as input to PCA are shown in Figure 5. In the top row, the three most significant eigenvectors are displayed for each acquisition condition. The 10 kHz SERDS difference principal components exhibit the highest signal quality. The ability to differentiate the two samples from each other is conveyed in the bottom row of the figure where the eigenvalues of PC1 are plotted against those of PC2 along with 95% confidence intervals. The 10 kHz data sets show a very clear ability to differentiate between the two plastic materials. (PLS-DA yielded specificity = 1 and sensitivity = 1 for both PS and PTFE, see Table II.) The 1 kHz data shows somewhat lower differentiation capability with data points from each sample being partially overlapped (PTFE specificity = 0.7, PTFE sensitivity = 0.9, see Table II) and the conventional SERDS read-out with 5.4 Hz yields strongly diminished ability to separate the two plastics from each other under these severe background conditions (PTFE specificity = 0.6, PTFE sensitivity = 0.8; see Table II).

**Table II.** Results of the partial least squares discriminant analysis (PLS-DA) in terms of sensitivity and specificity of the prediction model differentiating between PTFE and PS based on the measurements for the three read-out modalities.

PLS-DA	10 kHz CS		1 kHz CS		Conventional 5.4 Hz	
	PTFE	PS	PTFE	PS	PTFE	PS
Sensitivity (CV)	1.00	1.00	0.90	0.70	0.80	0.60
Specificity (CV)	1.00	1.00	0.70	0.90	0.60	0.80

Similar analysis was also performed on the reconstructed SERDS data sets and the results are shown in Figure S5 (Supplemental Material). Although somewhat lower differentiation performance was obtained with the reconstructed SERDS data sets overall compared with the corresponding SERDS difference data sets, a generally similar trend was observed. The 10 kHz charge-shifting mode showed the best differentiation performance followed by 1 kHz charge-shifting variant and complete inability to differentiate the samples exhibited by the conventional 5.4 Hz SERDS CCD read-out. The generally lower performance of the reconstructed data set in comparison with the SERDS difference data sets is ascribed to the reconstructed data sets containing additional distortion artefacts stemming from residual baseline modulations not fully removed by the applied polynomial baseline correction. These artefacts can partially mask chemical information contained in the spectra making it more difficult for the multivariate analysis (e.g., PCA, PLS-DA) to extract this information for differentiation between the two investigated plastic species. As such using the SERDS difference data set which contains the least artefact polluted, i.e., most pure, chemical information yields in our case the most satisfactory results, when fast spectral acquisition in charge-shifting mode at 10 kHz is applied.

### Quantification of Chemical Components

In many analytical applications, it is important not only to determine the chemical identity of an analyte but also its relative quantity in a matrix. Therefore, the performance of the charge-shifting read-out was also tested in this scenario using a vial containing varying mixtures of ethanol:methanol aiming at determining their actual concentrations. The data were analyzed with PLS regression using different prediction and calibration data sets, as described in the experimental section. In these experiments, the glass vial containing the solutions, which represents a fluorescent object (non-optical grade glass was used), was additionally overpainted with a highly fluorescent agent in an irregular manner to form a heterogeneous coating of the vial. The vial was then moved up and down in front of the detection system by hand to induce rapidly evolving fluorescence backgrounds. In this set of experiments, the room light was switched off and an equivalent integration time of 5 s was used for all three investigated detection modalities (Table I).

Typical SERDS difference spectra obtained for the 50% ethanol:50% methanol solution are shown in Figure S6a (Supplemental Material). For each of the three acquisition modalities, only three representative spectra are shown for clarity: the highest quality spectrum (red line, with the lowest background distortion), a medium distorted spectrum (black line) and the most distorted spectrum (blue line). As seen previously, the conventional 5.4 Hz SERDS CCD read-out resulted in the highest distortion of the obtained spectral background. The 1 kHz charge-shifting SERDS modality was considerably better, and the 10 kHz charge-shifting read-out exhibited the most stable spectral baseline indicating, as previously observed, that a sufficient rate of sampling was achieved for the concerned application.

**Figure 6.**
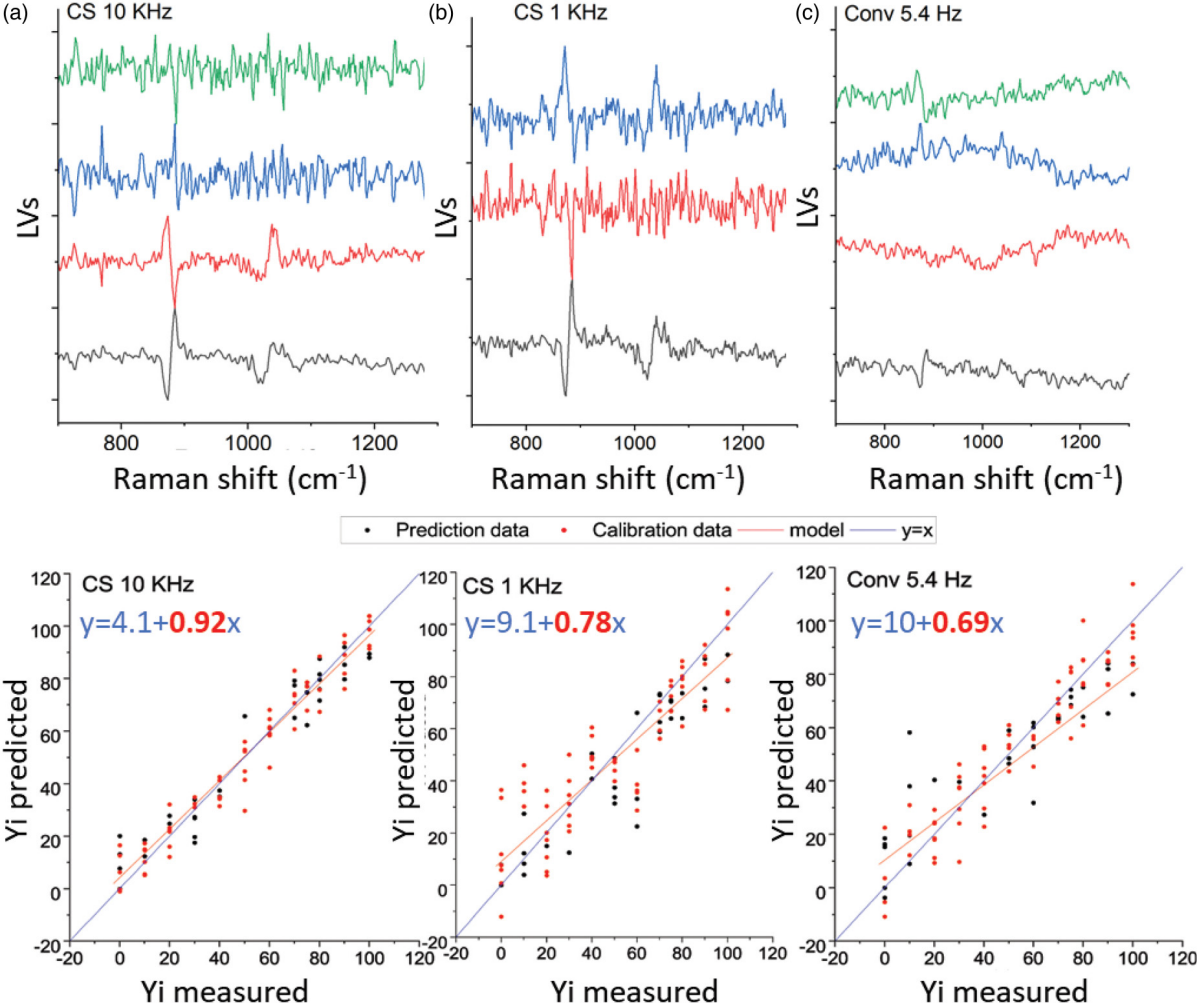
PLS prediction of methanol concentration with respect to ethanol. Top panel: LVs component.The number of components (LVs) was selected in correspondence with the minimum RMSEP value. Bottom panel: Predicted vs Measured methanol concentration (in %) for (a) 10 kHz charge-shifting (four LVs used), (b) 1 kHz charge-shifting (three LVs used), and (c) 5.4 Hz conventional (four LVs used). The equation show correspond to the model for the calibration data.

Figure S6b (Supplemental Material) shows SERDS difference spectra for each acquisition scenario using the E50M50 sample after seventh-order polynomial baseline subtraction. The conventional SERDS read-out is of the high level of noise with almost invisible analyte signatures. In contrast, the 1 kHz and 10 kHz charge-shifting SERDS methods show much better visibility of the most prominent analyte Raman signals (indicated by dashed lines). To determine the potential of the three different detection modalities for quantitative analysis, PLS was performed on the baseline corrected SERDS difference spectra with eight repetitions acquired for each concentration aiming for a prediction of the methanol concentration. The PLS analysis provides evidence that a decent performance in terms of quantification is still derivable from the 5.4 Hz conventional SERDS acquisition modality, despite the low visibility of chemical components in the spectra. In part, such a positive result is probably achieved due to the mixture being anti-correlated in composition meaning that the detection of any of the two components in any of the spectra also yields the concentration of the other, as expected from a binary mixture. Nevertheless, the errors of the prediction of the conventional SERDS read-out were comparatively higher (RMSEP = 15.7%), followed by the 1 kHz charge-shifting SERDS mode (RMSEP = 13.5%) with the 10 kHz read-out achieving again the highest accuracy of all (RMSEP = 8.4%) (see Figure 6). Table III lists the PLS results in terms of root mean squared error of calibration (RMSEC), root mean squared error of cross-validation (RMSECV) and root mean squared error of prediction (RMSEP). The 10 kHz regression also exhibited the lowest intercept value out of all indicating a higher model fidelity (see Figure 6).

**Table III.** PLS results in terms of the root mean squared error of calibration (RMSEC), root mean squared error of cross-validation (RMSECV), and root mean squared error of prediction (RMSEP) on the processed SERDS difference spectra of the 12 selected concentration of ethanol versus methanol mixtures.

	CS 10 kHz	CS 1 kHz	Conventional 5.4 Hz
RMSEC (%)	6.9	10.1	11.8
RMSECV (%)	11.8	18.7	30.4
RMSEP (%)	8.4	13.5	15.7

Finally, we also looked at the ability of the detection modalities to differentiate the mixtures from each other (qualitative analysis). In this case, we used two pure components: vials containing 100% of ethanol and 100% of methanol (i.e., E100M0 and E0M100). The result of this analysis is shown in Figure S8 (Supplemental Material, PCA and PLS-DA). This time much larger difference between the quality of the data from the three read-out modalities was present. The conventional SERDS read-out was in this case unable to differentiate the samples from each other (specificity = 0.88, sensitivity = 0.63 on methanol component with PLS-DA), the 1 kHz charge-shifting modality performed reasonably well (specificity = 0.88, sensitivity = 1 on methanol component with PLS-DA) and 10 kHz was even better (sensitivity and specificity = 1 on methanol component with PLS-DA), in line with the above results obtained for the two plastics. The much lower performance of the conventional 5.4 Hz read-out with the differentiation of solutions compared with the quantification application is ascribed to the need to detect each component in individual spectra in differentiation application, unlike in the quantification where the detection of one substance yields the concentration of the other substance due to the anti-correlation of the components in terms of their concentrations.

### Potential Future Directions

A natural next evolution of the charge-shifting concept could be the incorporation of the mask directly into the CCD chip in its lithographic fabrication process enabling perfect masking of unrestricted number of rows including every alternative row. This would potentially enable even faster acquisition rates as the charge would have to be moved only one row up and down. Such alternative row masking exposing only every second row was attempted in this study using a mask in front of our spectrometer but was not possible due to imaging aberrations within the spectrograph system preventing evenly and sharp imaging of the mask from the slit of the spectrograph onto the CCD chip. These limitations led us to use a mask blocking and transmitting every eight adjacent channel pixels (26 μm pixel size) where adequately accurate imaging was still achievable. Direct implementation of the mask on-chip could overcome this challenge, potentially enabling charge-shifting frequencies theoretically of up to 80 kHz.

## Conclusion

We have demonstrated an advanced charge-shifting CCD SERDS system capable of operating at speeds of up to 10 kHz. This is 10-fold faster than possible with our earlier charge-shifting variant and over 1000-fold faster than possible with conventional SERDS CCD read-out. The performance of the system was characterized on highly challenging samples exhibiting high level of dynamically evolving fluorescence. The 10 kHz charge-shifting read-out modality yielded the best performance in all the tested applications comprising differentiation of two analyzed solid plastic samples from each other and quantification of binary alcohol mixtures. Such fast-evolving backgrounds could be encountered in several real-world scenarios including those induced by natural fluorescence bleaching (in particular in Raman microscopy), ambient light condition changes or sample or instrument movement during acquisition over heterogeneous samples. Other scenarios include monitoring rapidly evolving Raman signals themselves in presence of largely static background signals such as in situations where a heterogeneous sample is rapidly moving in front of a detection system (e.g., on a conveyor belt) in presence of static ambient light. In general, it is worth noting that no benefit from using the technique would be accrued in situations where both the Raman signal and fluorescence backgrounds are static or dynamically evolving but their relative intensity ratio remains unchanged, as for example due to laser power fluctuations. Potential practical applications of the technique include highly accurate Raman microscopic mapping of complex matrices such as biological tissues in disease diagnosis or Raman measurements in case of impure samples and under ambient light. It should also be noted that the CCD charge-shifting method demonstrated here can be applied in other, non-SERDS areas of Raman spectroscopy not only for mitigating dynamic fluorescence or ambient light backgrounds but also in lock-in-like detection schemes to recover modulated signals. Equally, its applicability can further be extended to other optical sensing applications involving CCD detectors where the collection of two contrasting signals at fast acquisition rates is desired.

## Supplemental Material

sj-docx-1-asp-10.1177_00037028231167441 - Supplemental material for 10 kHz Shifted-Excitation Raman Difference Spectroscopy with Charge-Shifting Charge-Coupled Device Read-Out for Effective Mitigation of Dynamic Interfering Backgrounds Click here for additional data file. Supplemental material, sj-docx-1-asp-10.1177_00037028231167441 for 10 kHz Shifted-Excitation Raman Difference Spectroscopy with Charge-Shifting Charge-Coupled Device Read-Out for Effective Mitigation of Dynamic Interfering Backgrounds by Sara Mosca, Kay Sowoidnich, Megha Mehta, William H. Skinner, Benjamin Gardner, Francesca Palombo, Nicholas Stone and Pavel Matousek in Applied Spectroscopy
