# Supplementary material for: 10 kHz Shifted-Excitation Raman Difference Spectroscopy with Charge-Shifting Charge-Coupled Device Read-Out for Effective Mitigation of Dynamic Interfering Backgrounds
Source: Appl Spectrosc. 2023 Apr 25;77(6):569–82. doi: 10.1177/00037028231167441 (PMC10331517; doi:10.1177/00037028231167441)
Supplement: sj-docx-1-asp-10.1177_00037028231167441 - Supplemental material for 10 kHz Shifted-Excitation Raman Difference Spectroscopy with Charge-Shifting Charge-Coupled Device Read-Out for Effective Mitigation of Dynamic Interfering Backgrounds [file sj-docx-1-asp-10.1177_00037028231167441.docx]

**SUPPLEMENTAL MATERIAL**

**10 kHz Shifted-Excitation Raman Difference Spectroscopy with Charge-Shifting Charge-Coupled Device Read-Out for Effective Mitigation of Dynamic Interfering Backgrounds**

Sara Mosca^1^, Kay Sowoidnich^2^, Megha Mehta^3^, William Skinner^3^, Benjamin Gardner^3^, Francesca Palombo^3^, Nicholas Stone*^3^, Pavel Matousek*^1^

1. Central Laser Facility, Research Complex at Harwell, STFC Rutherford Appleton Laboratory, UKRI, Harwell Campus OX11 0QX, United Kingdom

2. Ferdinand-Braun-Institut, Leibniz-Institut für Höchstfrequenztechnik, Gustav-Kirchhoff-Str. 4, 12489 Berlin, Germany

3. Department of Physics and Astronomy, University of Exeter, Exeter EX4 4QL, United Kingdom

* Corresponding author emails: pavel.matousek@stfc.ac.uk, N.Stone@exeter.ac.uk

**Table of content:**

S1: Schematic of the masks and contrast 2

S2: Reference Raman Spectra of standard samples 3

S3: SERDS difference and reconstructed spectra and of PS in three read-out modalities 3

S4: Effect of the number of cycles on the CS process 4

S5: PCA results on SERDS-SORS reconstructed spectra (PS, PTFE) 5

S6: SERDS difference spectra obtained for the 50% ethanol:50% methanol solution 6

S7: PLS results on SERDS-SORS reconstructed spectra (ethanol: methanol %) 7

S8: PCA and PLS-DA results on SERDS-SORS difference spectra (E100M0 and E0M100) 8

**S1**: Schematic of the masks and contrast


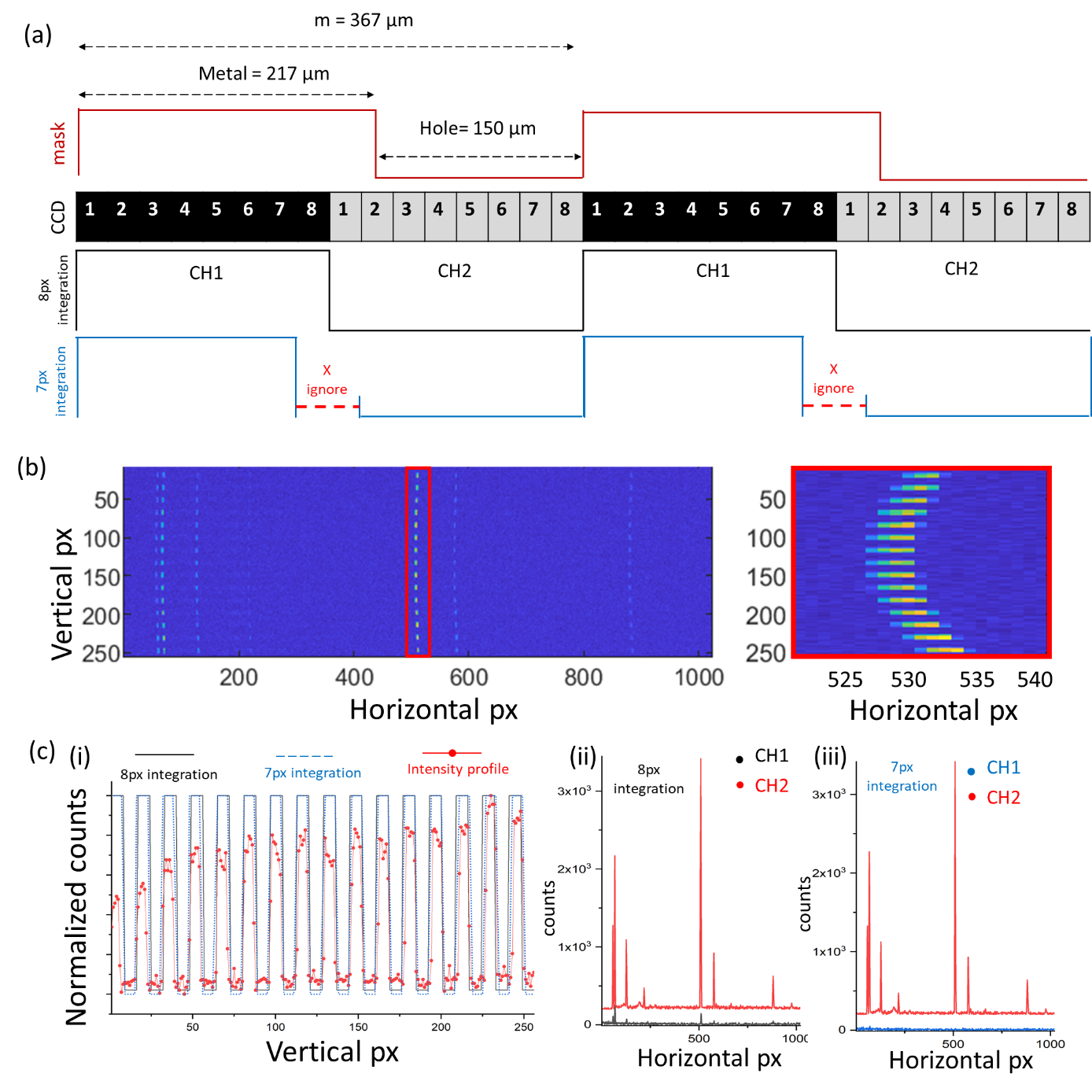


**Figure S1:** (a) Schematic of the metal mask (red rectangle) and its dimension with respect to the CCD (numbering pixels to highlight the spatial frequency of 8 px - progressive number 1 to 8). The rectangle below shows two possible integration patterns for extracting the CS spectra from the 2D image. Black rectangle: width = 8 px and spatial frequency = 8 px. Blue rectangle: width = 7 px and spatial frequency = 8 px. (b) 2D image readout in the conventional mode with room light ON shows the mask patterns on the CCD (acquisition time 5 s) (c) Intensity profile (i) obtained by integrating vertically the intensity in the red rectangle shown in (b). (ii-iii) spectra of the two channels CH1 and CH2 (vertically offset for clarity) obtained using (ii) the 8 px integration fix pattern and (iii) the 7 px integration pattern. The resulting contrast of the mask, defined as: CH1(OFF)/CH2(ON), is (ii) C (8px) = 4.5% and (iii) C(7px) = 0.6 %.

**S2:** Reference Raman Spectra of standard samples


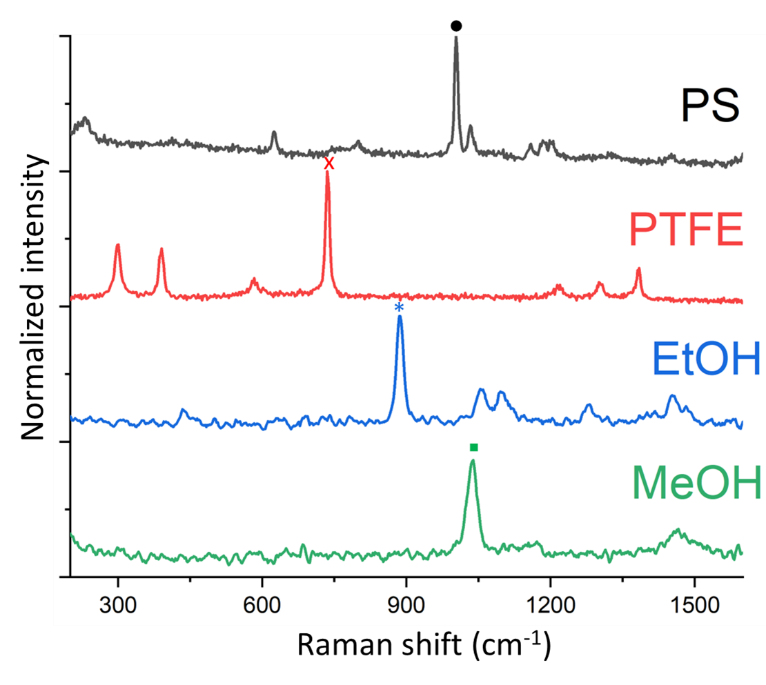


**Figure S2:** Reference Raman spectra of samples used in this study. The spectra were collected at L1 excitation wavelength (λ_1_ = 829.40 nm) with a zero spatial offset. Solid marks highlight the main Raman band of each material, from bottom to top: methanol (green line, filled square 1040 cm^-1^), ethanol (blue line, asterisk 887 cm^-1^), PTFE (red line, cross 734 cm^-1^), polystyrene (black line, circle 1003 cm^-1^).

**S3**: SERDS difference and reconstructed spectra and of PS in three read-out modalities

**
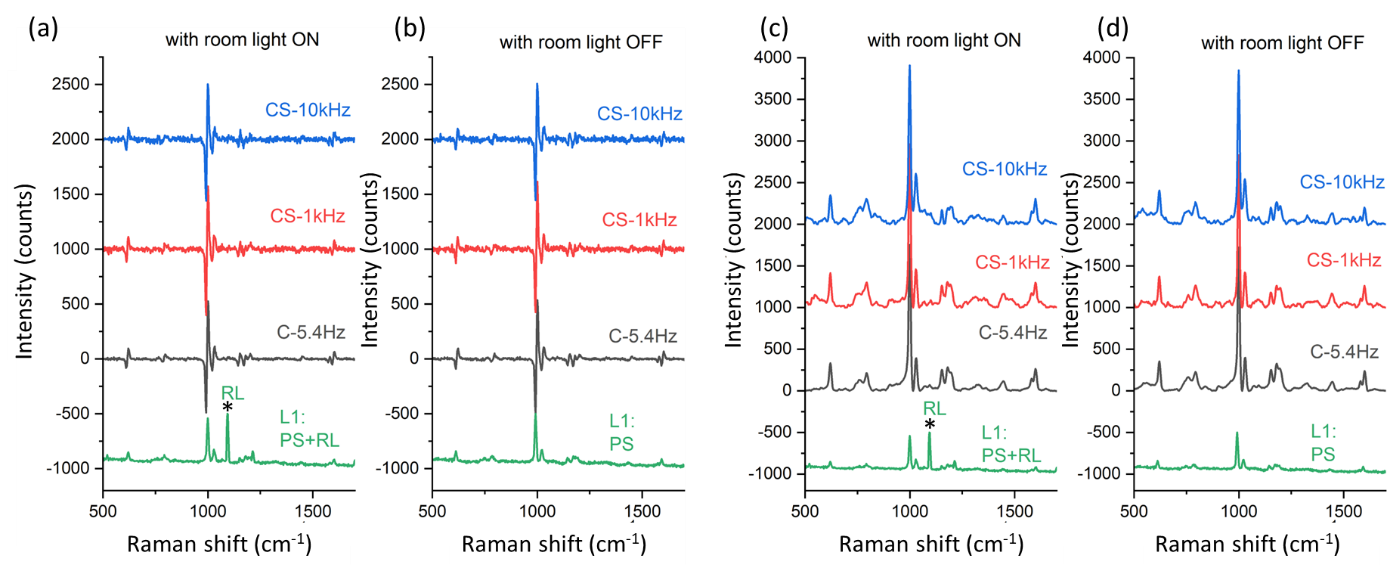
Figure S3:** (a-b) SERDS difference spectra of PS sample collected with the three read-out modalities using an equivalent overall acquisition time of 5 s and zero spatial offset (a) with and (b) without room light (RL). (c-d) Reconstructed SERDS spectra (c) with and (d) without room light. In each panel, the bottom green line shows the respective Raman spectra collected with a single excitation wavelength (L1). The band highlighted with the asterisk is the most intense room light feature in the investigated spectral region.

**S4:** Effect of the number of cycles on the CS process.


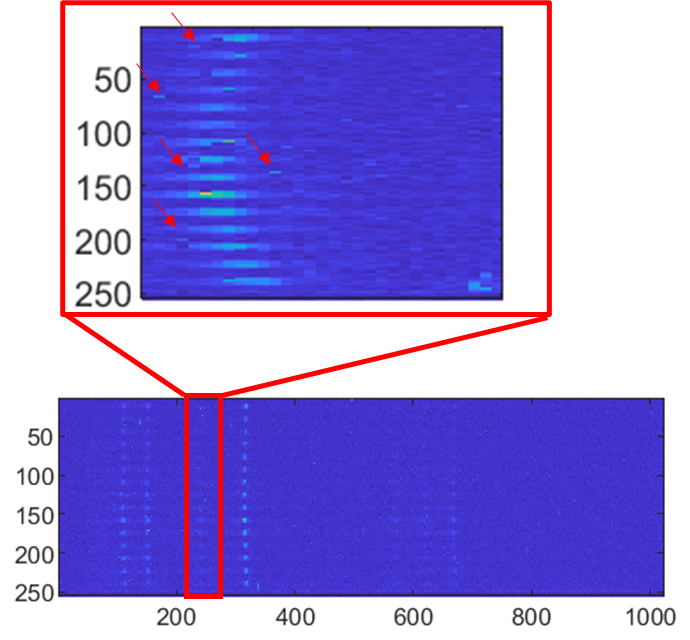


**Figure** **S4:** 2D image readout of CS method using 10 kHz and 50,000 cycles. The measurement was acquired on the PTFE sample with only one laser ON (L1) and in the presence of room light. The top zoom panel show the 2D intensity distribution around the PTFE main Raman peak at 734 cm^-1^. Red arrows highlight charge transfers into the wrong channels.

**S5:** PCA results on SERDS-SORS reconstructed spectra (PS, PTFE).
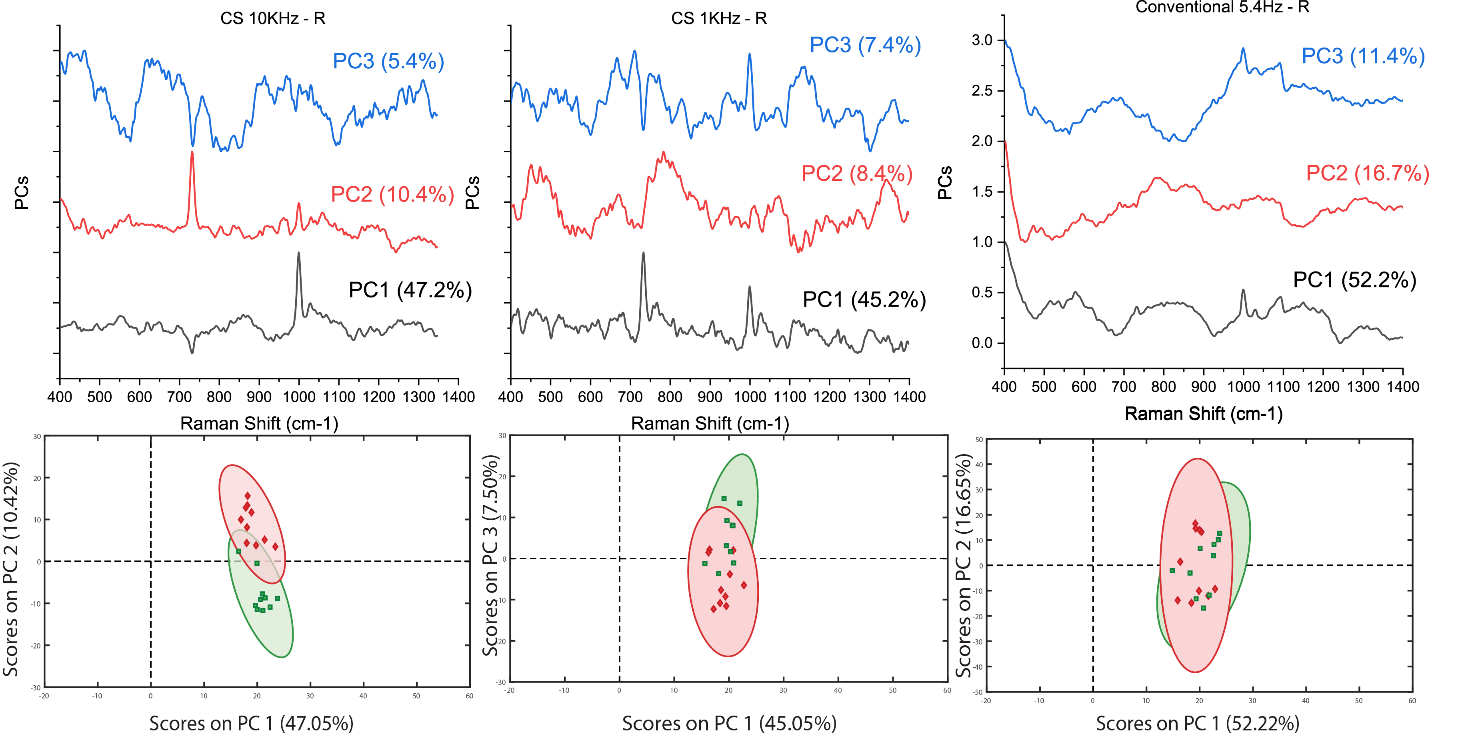


**Figure S5:** PCA results on SERDS-SORS reconstructed spectra of PS and PTFE in the presence of a strong heterogeneous fluorescent label signal from the top layer. From the left to the right are displayed the results for the three investigated read-out modalities. Top panel: PCA Loadings, Bottom panel: PCA Biplots.

**S6**: SERDS difference spectra obtained for the 50% ethanol : 50% methanol solution

**
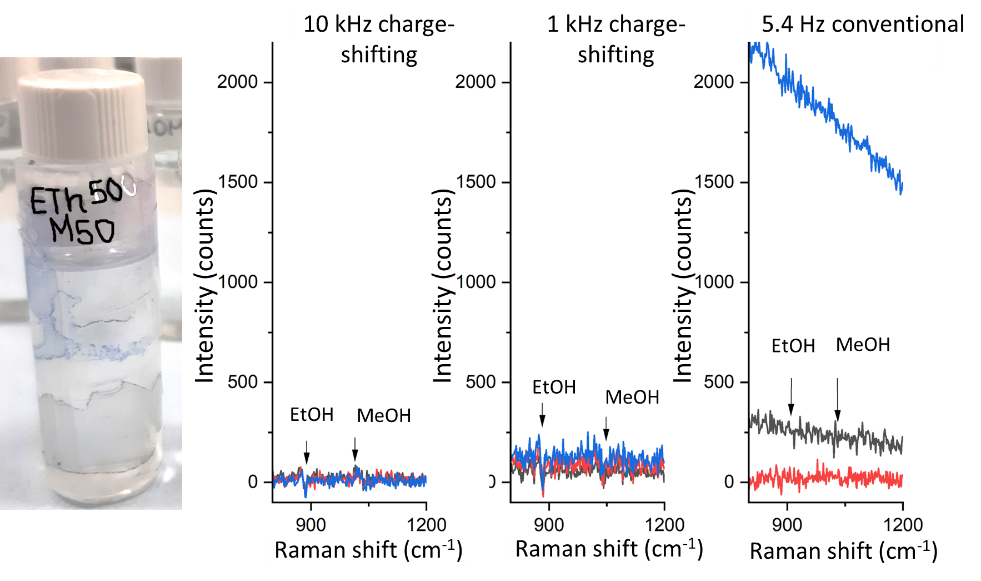
**

(b)

(a)


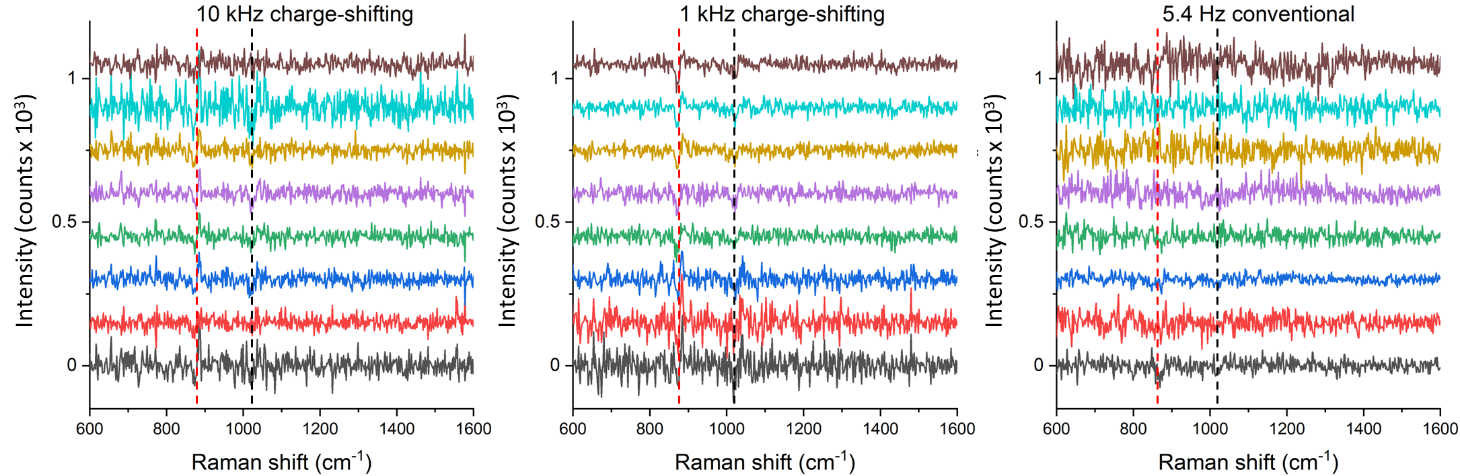


**Figure S6:**  (a) Vial containing a fluorescent agent and 50% ethanol:methanol solution (E50M50). Three representative raw SERDS difference spectra (without baseline subtraction) for the 50% ethanol:methanol mixture are shown. (b) E50M50 SERDS difference dataset made of 8 repetitions. Spectra are shown after baseline removal (using a 7^th^ order polynomial). Vertical dashed lines indicate the strongest Raman signals of target analytes methanol (black) and ethanol (red)**.**

**S7:** PLS results on SERDS-SORS reconstructed spectra (ethanol: methanol %).


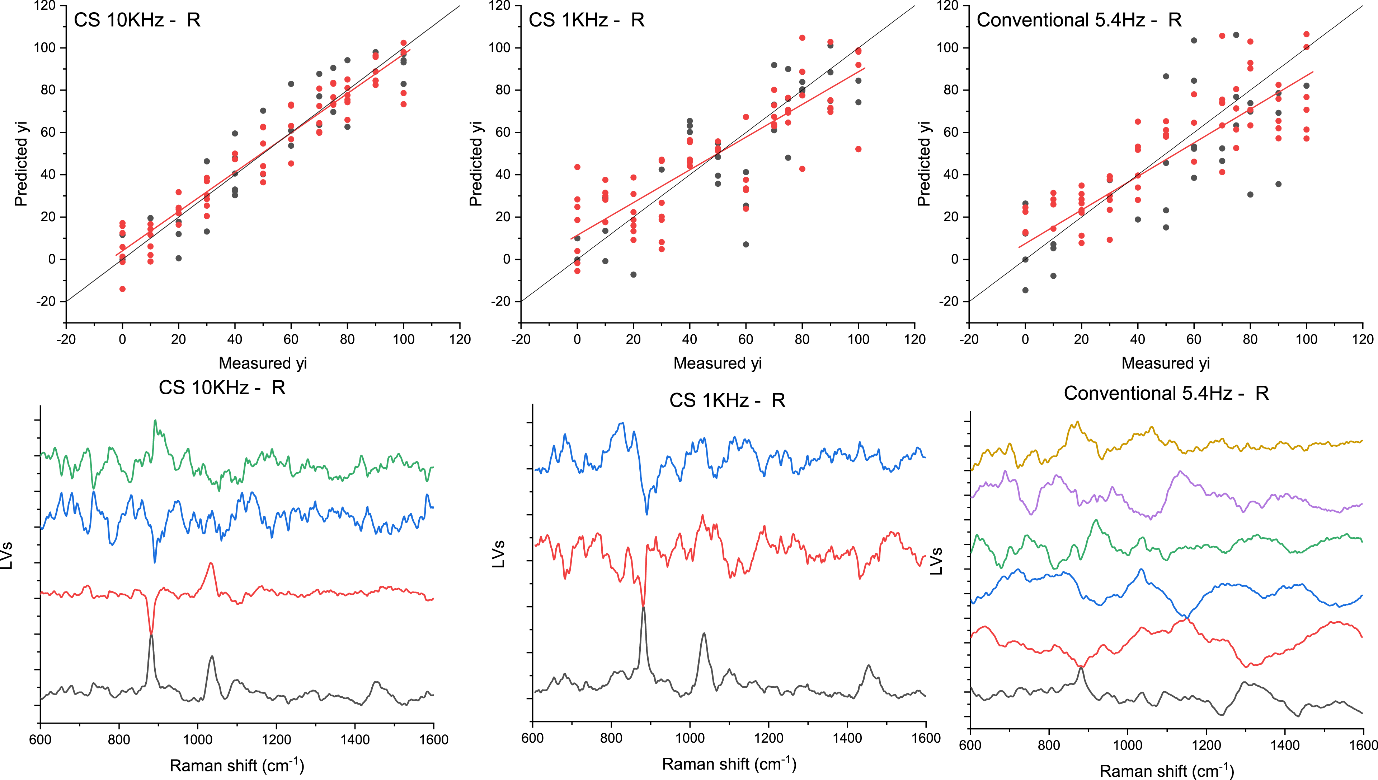
 **Figure S7:** PLS prediction of 12 chosen methanol concentrations with respect to ethanol. The table summarizes the PLS results in terms of root mean squared error of calibration (RMSEC), root mean squared error of cross-validation (RMSECV) and root mean squared error of prediction (RMSEP). Top panel: Measured versus Predicted concentration for 10 kHz (4 LVs used), 1 kHz (3 LVs used) and 5.4 Hz (6 LVs used) (left to right). The number of components (LVs) was selected in correspondence with the minimum RMSEP value. Bottom panel: LV components.

**Table S7:** PLS results in terms of the root mean squared error of calibration (RMSEC), root mean squared error of cross-validation (RMSECV) and root mean squared error of prediction (RMSEP) on the processed reconstructed SERDS spectra of 12 chosen concentration ethanol vs methanol mixtures.

|  | **CS – 10 kHz** | **CS – 1 kHz** | **Conventional 5.4 Hz** |
| --- | --- | --- | --- |
| RMSEC (%) | 8.96 | 15.09 | 16.79 |
| RMSECV (%) | 12.09 | 21.66 | 27.21 |
| RMSEP (%) | 12.06 | 17.54 | 25.70 |

**S8:** PCA results on SERDS-SORS difference spectra (E100M0 and E0M100).
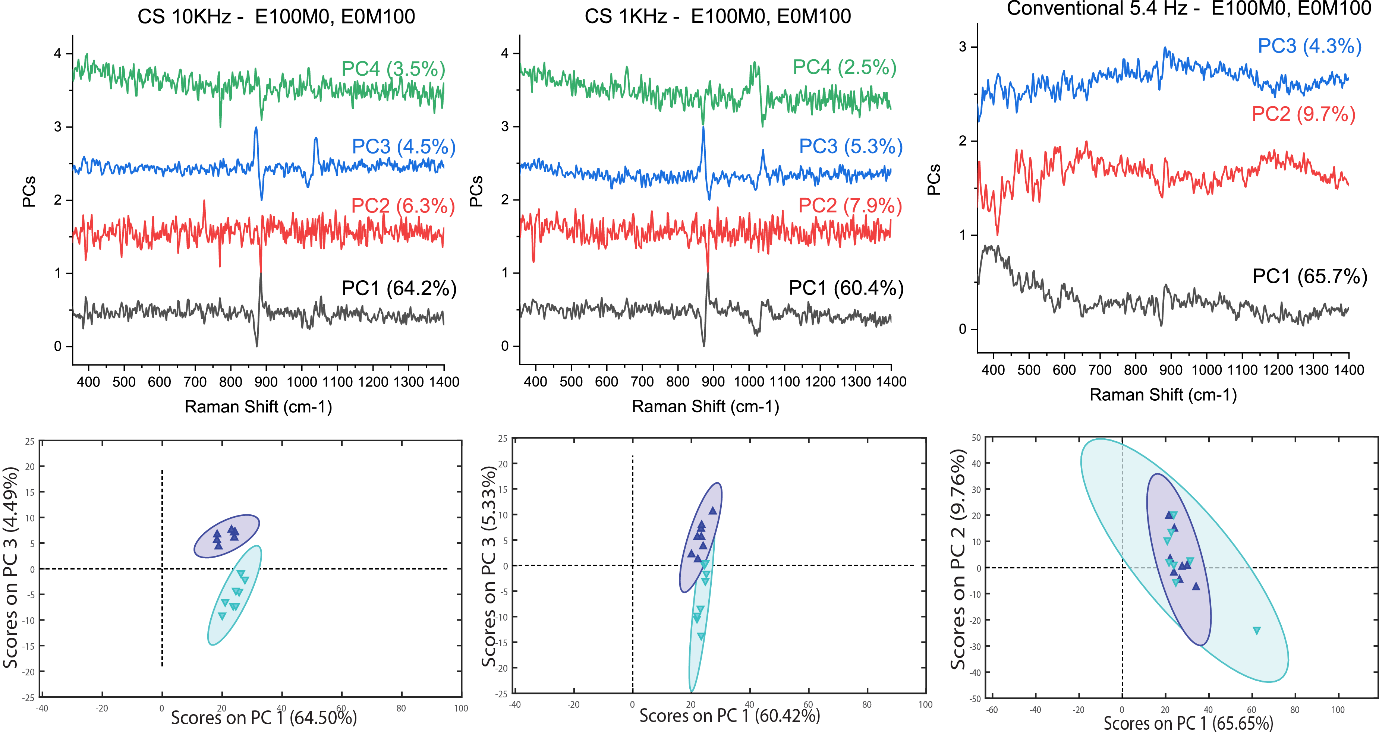


**Figure S8:** PCA results on SERDS-SORS difference spectra of 100% ethanol vs 100% methanol (E100M0 and E0M100) dataset in the presence of a strong heterogeneous fluorescent label signal from the top layer. From the left to the right are displayed the results for the three read-out modalities. Top panel: PCA Loadings, Bottom panel: PCA Biplots.

**Table S8: PLS-DA –** Results of the Partial Least Squares Discriminant Analysis performed on the ethanol 100% and methanol 100% measurements for the three read-out modalities.

| **PLS-DA** | **10 kHz CS** | | **1 kHz CS** | | **CONV 5.4 Hz** | |
| --- | --- | --- | --- | --- | --- | --- |
|  | **MeOH** | **EtOH** | **MeOH** | **EtOH** | **MeOH** | **EtOH** |
| Sensitivity (CV) | 1.000 | 1.000 | 0.88 | 1.000 | 0.88 | 0.63 |
| Specificity (CV) | 1.000 | 1.000 | 1.000 | 0.88 | 0.63 | 0.88 |

PLS-DA analysis confirms the trend observed in the PCA. The model based on the conventional SERDS readout was in this case less capable to differentiate between ethanol and methanol samples from each other with a sensitivity and specificity on the ethanol component equal to 0.63 and 0.88 respectively, the 1 kHz charge-shifting modality performed reasonably well (specificity = 1, sensitivity = 0.88) and 10 kHz readout led to the best prediction model with a sensitivity and specificity equal to 1.
